# 2245. Appropriate use of antibiotics in patients with osteomyelitis due to decubitus ulcers

**DOI:** 10.1093/ofid/ofad500.1867

**Published:** 2023-11-27

**Authors:** Jaraad Dattadeen, Sadaf Aslam

**Affiliations:** University of South Florida, Tampa, Florida; University of South Florida Morsani College of Medicine, Tampa, Florida

## Abstract

**Background:**

Decubitus ulcers are a costly and challenging problem in the healthcare system, with an estimated 2.5 million pressure ulcers treated annually in the United States at a cost of $11 billion. Patients with decubitus ulcers may present with evidence of infection or sepsis, but the source is rarely the ulcer itself. Recent guidelines recommend evaluating patients for curative management before pursuing further testing or empiric antimicrobial treatment. This study aimed to assess the appropriateness of antibiotic usage in patients admitted to the hospital with decubitus ulcers, and to determine the rate of appropriate antibiotic use.

**Methods:**

This study involved a retrospective review of 1678 patients records with decubitus ulcers to assess the appropriate use of antibiotics during their inpatient stay. Appropriateness was determined by one of three criteria. The 1st criteria included patients who met systemic inflammatory response syndrome with suspected infection from osteomyelitis; 2nd criteria included patients who received antibiotics due to another infection (pneumonia, urinary tract infection or bacteremia) and 3rd criteria included use of antibiotics as a bridge therapy to surgical intervention (flap closure or Incision and Drainage). The rate of consultation for infectious disease and plastic surgery was also investigated.

**Results:**

Of the total 1678 patients, the median age was 70 yrs (IQR=21), of that 51 % were males. Based on the criteria for appropriateness, there were a significant difference in gender (p= 0.000) females were more likely to receive inappropriate antibiotics (Table 2). Similarly, the rate of inappropriate use of antibiotics was 15.9% with ID consults as compared with 84.1% who didn’t receive ID consult (p=0.000) as seen in table 1.

- 1000+ embedded vimeo player sites supported
- Support video downloading in more resolutions e.g. 4k, 2k, 1080p, etc.
- Support for downloading private videos
- Support for extracting audio
- Support for downloading subtitles
- Unlimited downloads

What is the payment method you want?

- 1000+ embedded vimeo player sites supported
- Support video downloading in more resolutions e.g. 4k, 2k, 1080p, etc.
- Support for downloading private videos
- Support for extracting audio
- Support for downloading subtitles
- Unlimited downloads

What is the payment method you want?

- 1000+ embedded vimeo player sites supported
- Support video downloading in more resolutions e.g. 4k, 2k, 1080p, etc.
- Support for downloading private videos
- Support for extracting audio
- Support for downloading subtitles
- Unlimited downloads

What is the payment method you want?

- 1000+ embedded vimeo player sites supported
- Support video downloading in more resolutions e.g. 4k, 2k, 1080p, etc.
- Support for downloading private videos
- Support for extracting audio
- Support for downloading subtitles
- Unlimited downloads

What is the payment method you want?

**Figure:**
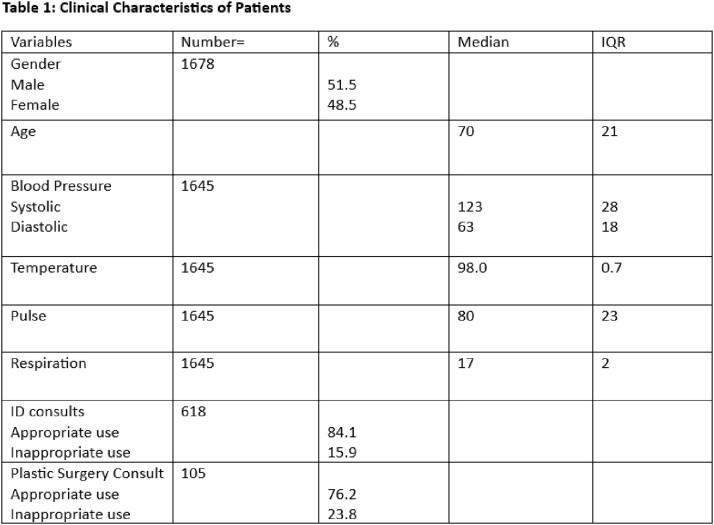

**Figure:**
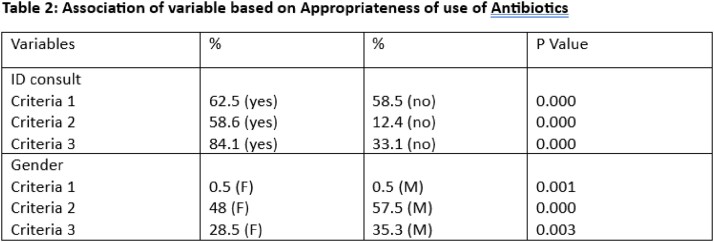

**Conclusion:**

This study contributes to a better understanding of appropriate antibiotic usage in patients with decubitus ulcers. The findings favor the importance of ID consults in the appropriate use of antibiotics. Additionally, there may be some underlying bias with prescribing antibiotics to females compared to males. Thus, the study provides clinicians with tools in order to make evidence-based decisions on the management of these patients, and for healthcare systems to optimize resource allocation

**Disclosures:**

**All Authors**: No reported disclosures

